# TB Meningitis and TB Peritonitis: Abdominal Pseudocyst and VP-Shunt Link

**DOI:** 10.1155/2019/4893547

**Published:** 2019-04-28

**Authors:** Manzoor Ahmed, Essameldin Ali ElGamal, Anwar Ahmad, Muhammad Badar Zaman

**Affiliations:** ^1^Department of Radiology, Sheikh Khalifa Medical City, Abu Dhabi, UAE; ^2^Department of Neurosurgery, Sheikh Khalifa Medical City, Abu Dhabi, UAE; ^3^Department of Surgery, Sheikh Khalifa Medical City, Abu Dhabi, UAE

## Abstract

TB meningitis (TBM) carries high morbidity and mortality and is a relatively common extrapulmonary TB in the third world countries. TBM as thick exudative disease manifests on MRI and CT as nodular basal leptomeningitis, hydrocephalus, basal infarcts, and tuberculomas. Hydrocephalus is treated with ventriculoperitoneal shunting (VPS). Shunt malfunction and revision are common. We report a case of multidrug-resistant TBM with spinal involvement and dissemination of the disease via VPS causing TB peritonitis (TBP). TBP presented as a large abdominal pseudocyst around the catheter tip with shunt malfunction. There was no evidence for any other site of extra-CNS disease. TBP per se is relatively less common. This is the first case reporting VPS as a means of TB spread.

## 1. Introduction

TB is a well-known global health problem, specifically in the developing countries. Approximately one-fifth of the TB cases have extrapulmonary involvement, including TB meningitis (TBM). Approximately 50% of the patients with TBM become disabled or die. In Africa, adult TBM has a very high mortality rate (60%), which has not changed over several decades [1].

Tuberculous meningitis is typically caused by* Mycobacterium tuberculi* entering via a hematogenous route from the lungs (a primary site of infection). A key step in the pathogenesis of TBM is the development of caseating foci in the brain parenchyma and meninges, termed “rich foci.” These develop around the deposited bacteria during the bacteremic phase and later rupture into the subarachnoid space, causing meningitis. The most characteristic gross pathologic feature of TBM is meningeal inflammation and formation of thick gelatinous exudates in the basal parts of the brain [2]. This is reflected by thick nodular and cystic meningeal enhancement on contrast enhanced magnetic resonance imaging (MRI). Additional adverse effects are associated with the thick exudate, including hydrocephalus due to CSF pathway blockage and impaired drainage via ventriculoperitoneal shunts (VPS), requiring frequent shunt revisions. Shunt complications occur in approximately 32% of the cases [3, 4]. If medical treatment fails, VPS is the procedure of choice, preferred over endoscopic third ventriculostomy (ETV), based on clinical outcomes [3].

TB meningitis itself can theoretically be considered the source of infection spread and shunt complication in the peritoneal cavity, including pseudocyst formation. However, the literature surrounding this is controversial, with no definite reported cases of meningitis extension following VPS. It is also possible that the thick exudative may play a preventive role. Hence, it has not precluded surgeons from performing VPS in TBM-caused hydrocephalus. De novo TB peritonitis (TBP) is also rare. Takase* et al.* [5] reported primary TBP causing shunt malfunction in non-TB causes. Here, we report a rare case of multidrug-resistant TBM with intracranial and spine involvement, followed by spread via VPS into the peritoneal cavity, resulting in large pseudocyst formation due to TBP around the tip of the catheter, causing shunt dysfunction.

## 2. Case Report

**Figure 1:**
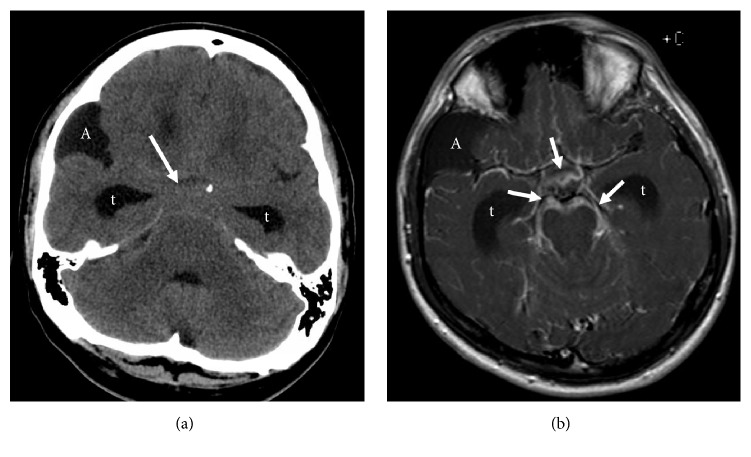
Axial noncontrast CT Head (a) and postcontrast T1 (b) MR images at the time of admission. Isodense basal leptomeningitis (arrow) on CT (a) with corresponding thick enhancement (arrows) on MRI (b). Hydrocephalus with dilated temporal horns (t). Incidental arachnoid cyst (A).

**Figure 2:**
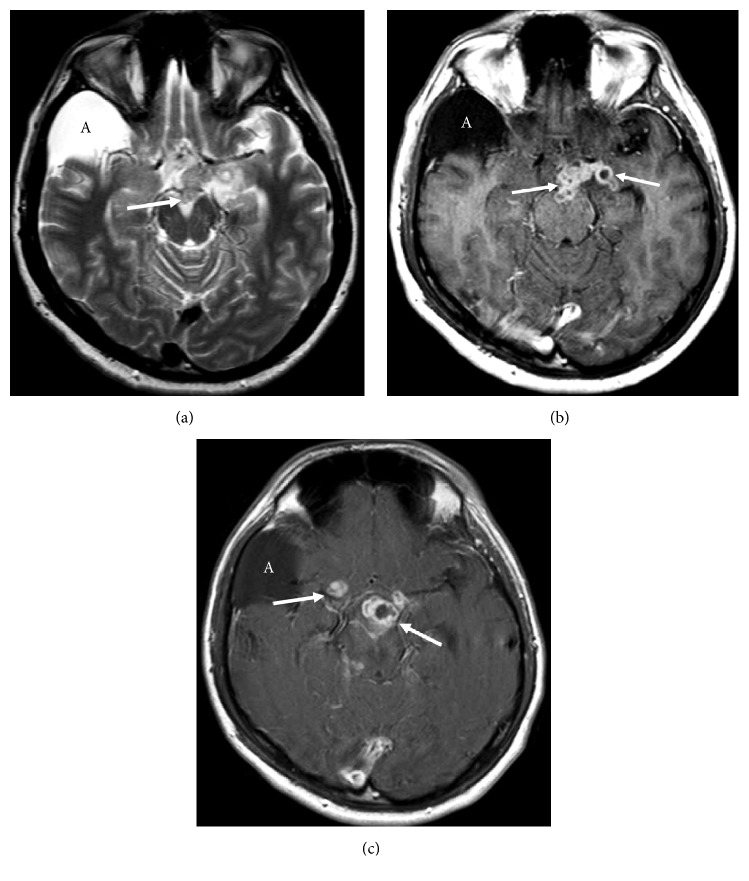
Axial FSE T2 (a) and postcontrast T1 (b and c) images at about 2 weeks of admission. T2 hypointense (a) nodular basal leptomeningeal lesions (arrows) with rim enhancement (b and c). Incidental arachnoid cyst (A).

An 18-year-old immune-competent male patient with no significant medical history was admitted to our hospital with 1-week history of headache, fever, and change in mental status suggestive of meningitis. The CSF analysis showed a high leucocyte count, lymphocyte predominance (92%), and high protein (0.92 g/L) and relative low glucose levels (4 mmol/L). Rapid TB PCR, HSV PCR, and India ink staining test results were negative. Chest imaging and sputum analysis did not reveal any positive findings. Initially, he was treated empirically for pyogenic meningoencephalitis. Follow-up computer tomography (CT) and MRI performed 2 weeks later showed hydrocephalus and basal meningeal disease with a pattern of cystic nodular enhancement (Figures 1 and 2) highly suggestive of TBM. At this time, the patient was also empirically treated with anti-TB medications (rifampicin, isoniazid, ethambutol, and pyrazinamide) supplemented with steroids. An external ventricular drain was placed, followed by conversion into a VPS. MRI of the spine revealed diffuse smooth dural thickening, representing nonspecific meningitis. The patient showed slow improvement and was discharged only on anti-TB medications.

**Figure 3:**
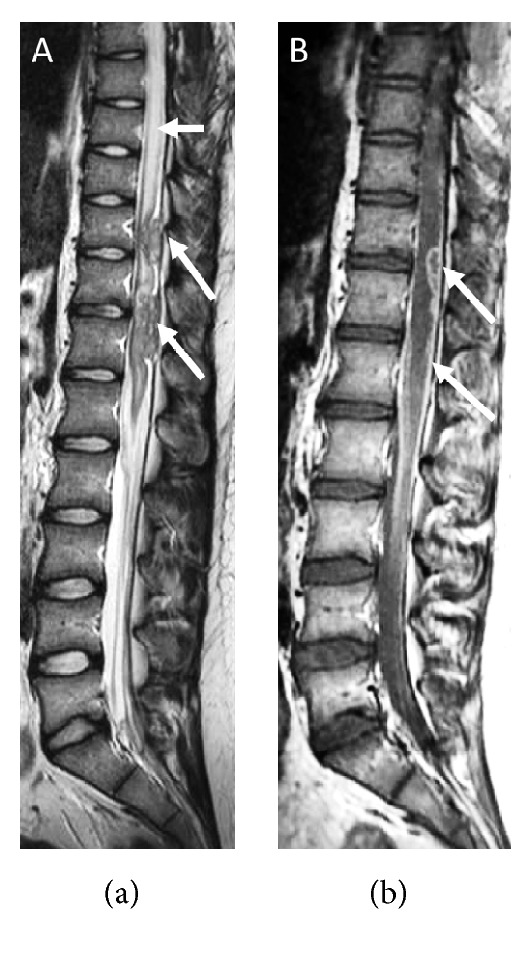
Sagittal FSE T2 (a) and postcontrast T1 (b) MR images of thoracolumbar spine at about 3 months of first admission. Spinal cord edema (Arrow 1). Diffuse nodular leptomeningeal and dural disease with ring enhancing abscesses (Arrow 2).

After approximately 3 months, the patient was readmitted with bilateral leg weakness. The MRI of the spine showed progressive diffuse dural thickening and enhancement with intradural thick-walled abscesses causing cord compression and edema (Figure 3). The patient underwent spinal decompression. Acid-fast bacilli (AFB) stains were negative and mycobacterial culture did not show growth. However, the patient was continued on first-line anti-TB therapy.

**Figure 4:**
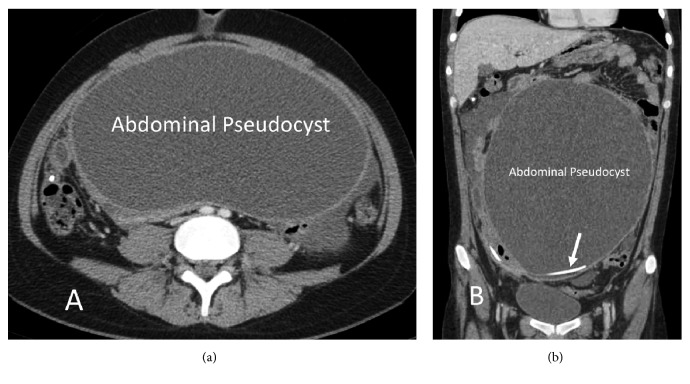
Axial (a) and coronal (b) noncontrast CT reformatted images at about 9 months after ventriculoperitoneal shunt placement. Large thick-walled abdominal pseudocyst with smooth walls. Note VP shunt catheter within the base of the cyst (arrow, b).

Approximately 6 months later, he presented with shunt dysfunction and an enlarging abdominal cystic mass related to the tip of the VPS catheter revealed by CT of the abdomen (Figure 4). Laparoscopy showed a large pseudocyst with local inflammatory changes. The pseudocyst was drained and resected and the VPS was removed. Finally, mycobacterium complex was isolated in culture from the cyst aspirates and a CSF shunt tap (mycobacterium complex included* M. tuberculosis*,* M. africanum*,* M. bovis*,* M. microti*, and* M. pinnipedii*). Multidrug-resistant TB was confirmed by a drug sensitivity test, given resistance to rifampicin and isoniazid and sensitivity to ethambutol and pyrazinamide. This led to modifying the regimen to a 7-drug therapy including moxifloxacin, pyrazinamide, ethambutol, ethionamide, and cycloserine (for at least 12 months), in addition to Amikacin and Linezolid (for 6 months). An external ventricular drain was inserted and then removed after passing the clamp-challenge test. Patient showed slow progressive clinical improvement with no new disease and stable appearance, specifically with respect to the nodular basal meningitis and ventricular caliber.

## 3. Discussion

Imaging plays a vital diagnostic role in TBM. Typical neuroimaging characteristics of TBM classically include leptomeningeal and basal cisternal enhancement and ventriculomegaly due to hydrocephalus, periventricular infarcts, and the presence of tuberculomas [6]. Our case showed basal meningitis complicated by communicating hydrocephalus followed by VPS dysfunction due to pseudocyst formation around the tip of the catheter.

In children, about two-thirds of patients with TBM develop hydrocephalus [3] with an unfavorable impact on prognosis [7]. The prevalence of hydrocephalus is much lower in adults [3]. In the majority of cases (>80%), hydrocephalus is communicating rather than obstructive [3]. Clinical grading is used to assess TBM. Medical management is the first line of therapy and includes steroids, dehydration agents like mannitol, and diuretics like frusemide and acetazolamide to reduce CSF production. However, there is evidence of better outcomes with early surgery, rather than waiting for a response to medical treatment [4]. The hydrocephalus in our case was diverted via VPS during the early course of the disease.

The thick exudative and gelatinous nature of the leptomeningeal inflammation is a high risk for shunt complications, warranting frequent revision [3, 4]. The typical mechanisms of shunt malfunction include breakage, blockage due to debris, adhesions, and pseudocyst formation. Furthermore, Ambekar* et al.* [8] showed high CSF protein content as a risk factor for shunt blockage and dysfunction. This makes our case unique and important, underscoring persistent TB infection (due to drug-resistant TB) as a cause of TB spread via VPS. We believe that the VPS likely provided a conduit for the elusive mycobacteria to spread down to the tip of the catheter in the abdomen, as there was no evidence of extra-CNS disease elsewhere in the body. It can be hypothesized that the pseudocyst formed before it was infested with mycobacteria and later provided a reservoir for mycobacterial growth. MRI performed approximately 2 weeks later clearly demonstrated gross intracranial basal CSF disease typical of TBM, later manifesting as spinal CSF disease. This indicated active CSF disease and represented a fertile source to transport the disease to directly connected spaces. Eventually, there was also isolation of mycobacterial complex from the CSF and pseudocystic contents simultaneously.

Placement of a VPS is generally advised in the acute stage of TBM if hydrocephalus is noncommunicating or following failed medical therapy in communicating hydrocephalus [4]. Reasonably successful outcomes have been found with direct VPS (as a first line therapy) even in advanced hydrocephalus (grade 3-4) [9]. Endoscopic third ventriculostomy has demonstrated variable success in TBM and is generally not advisable in patients during the acute stages of the disease [10, 11].

As discussed above, shunt malfunctions can manifest in the southern waters of the peritoneal cavity in the form of pseudocyst formation. Ascites may be associated with more abdominal symptoms; however, if a pseudocyst is present, it can result in shunt obstruction in approximately 60% of cases [12]. Several studies have reported abdominal pseudocyst formation following insertion of CSF shunts, since the first description by Harsh* et al*. in 1954 [13, 14].

Abdominal pseudocyst is an uncommon manifestation following VPS [15]. There are multiple predisposing factors associated with pseudocyst formation, including infection, multiple shunt revisions, obstruction or dislodgement, and foreign body reaction [16]. Hahn* et al.* [13] reported that infection was the most likely cause of pseudocyst formation (80%). They proposed infection as the presumed etiology for abdominal pseudocysts, as evidence of infection is confirmed in a small number of cases [17]. The infection rate ranges from 17 to 80%, with S. epidermidis and S. aureus identified as the most common pathogens [15, 18, 19]. In most cases, external drainage and antibiotics suffice to produce optimal clinical outcome [18]. Kariyattil* et al*. [12] reported that patients with abdominal pseudocysts had an average of approximately 3 shunt revisions compared to an average of less than 1 revision in a group with ascites without pseudocysts. Our case did not experience previous surgery or pyogenic infection. He presented with secondary TBP related to VPS and pseudocyst formation around the catheter tip. Tuberculous peritonitis* per se* does not typically cause pseudocyst formation, with no reported cases in the English literature. Notably, our patient showed no evidence of extra-CNS disease except localized peritoneal disease with an abdominal pseudocyst around the catheter tip, further suggesting spread via VPS.

Tuberculous peritonitis is a serious manifestation of this global disease. Like other extrapulmonary sites, the disease is imported via a hematogenous route. The risk factors for TBP include immunocompromised state, chronic kidney disease, and cirrhosis/liver disease. It constitutes about 30-60% of abdominal TB (other sites include the gastrointestinal tract and mesenteric lymph nodes [20]). An apparent risk factor is chronic kidney disease requiring continuous ambulatory peritoneal dialysis (CAPD) [21]. There are 4 described gross pathologic forms of TBP, including (1) wet-ascites, (2) fibrotic-fixed, (3) dry-plastic, and (4) mixed types. The diagnosis of TBP is usually delayed until subacute presentation [20]. The diagnosis of TBP requires ascitic fluid analysis for serum ascitic albumin gradient (SAAG) calculation, mycobacterial culture growth, peritoneal biopsy, laparoscopy, or laparotomy. CT imaging shows ascitic fluid to have higher attenuation values, diffusely thickened peritoneum, and soft tissue omental changes [22, 23].

Our case exemplifies a very rare scenario in the following aspects: (1) the occurrence of direct dissemination of TBM through VPS catheter causing TBP and manifesting clinically and on imaging in the form of shunt malfunction due to TB pseudocyst formation, (2) CSF cultures showing multidrug-resistant TB strains, requiring modification of the anti-TB treatment, which likely contributed to the dissemination of TB into the peritoneal cavity, and (3) extensive CNS disease, but no extra-CNS disease, except localized TBP, signifying a VPS link between TBM and TBP. Certainly, early institution of appropriate rather than empirical anti-TB therapy would have prevented relapse of the disease in the CNS and peritoneal cavity.
